# Association of maternal uric acid and cystatin C serum concentrations with maternal and neonatal cardiovascular risk markers and neonatal body composition: The Ulm SPATZ Health Study

**DOI:** 10.1371/journal.pone.0200470

**Published:** 2018-07-19

**Authors:** Dietrich Rothenbacher, Stefanie Braig, Chad A. Logan, Gertrud Feike, Miriam Müller, Wolfgang Koenig, Frank Reister, Jon Genuneit

**Affiliations:** 1 Institute of Epidemiology and Medical Biometry, Ulm University, Ulm, Germany; 2 Department of Internal Medicine II—Cardiology, University Medical Center Ulm, Ulm, Germany; 3 Deutsches Herzzentrum München, Technische Universität München, Munich, Germany; 4 DZHK (German Centre for Cardiovascular Research), partner site Munich Heart Alliance, Munich, Germany; 5 Department of Gynecology and Obstetrics, University Medical Center Ulm, Ulm, Germany; 6 Member of ‘In-FLAME’ the International Inflammation Network, World Universities Network (WUN), West New York, United States of America; Holbæk Hospital, DENMARK

## Abstract

**Purpose:**

In utero exposure to cardiometabolic risk factors may determine health related outcomes at birth and in later life. The aim of this analysis was to describe the relationship of maternal serum uric acid (SUA) and cystatin C with maternal and neonatal cardiometabolic risk markers and with birth weight and risk of small-for-gestational age (SGA) as well as large-for gestational age (LGA).

**Material and methods:**

In the Ulm SPATZ Health Study, 934 singleton newborns and their mothers were recruited during their hospital stay in the University Medical Center Ulm between 04/2012 and 05/2013 (overall response 49%). The association between SUA and cystatin C (both in quartiles and as continuous measures) with risk for SGA as well as with LGA was quantified by means of multivariable logistic regression.

**Results:**

Overall, n = 885 mother-newborn pairs were included in the final analysis. Most of the mothers were of German nationality (85%) and were between 26 and 35 years of age at delivery (69%). Maternal SUA was associated with maternal age, body mass index, alcohol consumption and history of hypertension as well as with many other maternal and neonate cardiovascular risk markers. Cystatin C was associated with parity. No clear association of SUA with SGA and LGA was observed in fully adjusted models. However, cystatin C was negatively associated with SGA with an odds ratio (OR) of 0.35 (95% CI: 0.16–0.77; p for trend 0.04) comparing the top quartile vs. the bottom quartile and was positively associated with LGA with an OR of 5.92 (95% CI: 2.27–15.44; p for trend <0.0001) after adjustment for covariates.

**Conclusions:**

We found a positive association of cystatin C with birth weight and a clearly increased risk for LGA with maternal increased cystatin C values in a population with fairly normal renal function.

## Introduction

Many studies in adults have shown that uric acid is a risk factor for cardiovascular disease both in primary as well as secondary risk settings [1,2]. A relationship between uric acid and inflammatory markers and multiple cytokines is also well documented. Serum uric acid (SUA) also exhibits direct immune-modulating effects [3], implying a potential role for SUA in atherosclerosis and other cardiometabolic disorders characterized by low grade inflammation such as obesity, the metabolic syndrome, hypertension, diabetes, other inflammatory processes, and especially, with renal function.

SUA crosses the placenta and levels of SUA vary throughout pregnancy. In early pregnancy maternal serum levels decrease because of the uricosuric effects from estrogens and the increase of renal blood flow. During the third trimester SUA increases considerably and may reach high levels ranging from 238 to 298 μmol/L [4]. Higher levels of SUA have been associated with blockage of endothelial growth proliferation and a relationship to fetal growth has been suggested [5]. SUA has also been related to adverse outcomes in both the mother and the fetus in hypertensive pregnancy [6].

Renal function, as assessed by cystatin C levels, a reliable marker for renal function, in pregnancy may also be indicators of maternal cardiometabolic disease [7] affecting fetal health. Several studies described the relationship between maternal SUA, renal function and fetal outcomes [8–12] and results are controversial.

The aim of this analysis was to describe the relationship of maternal SUA and cystatin C with maternal and neonate cardiometabolic risk markers and with birth weight and neonatal body composition (i.e. risk of small-for-gestational age (SGA) as well as risk of large-for-gestational age (LGA)) within a birth cohort study.

## Material and methods

### Study design and population

**Fig 1:**
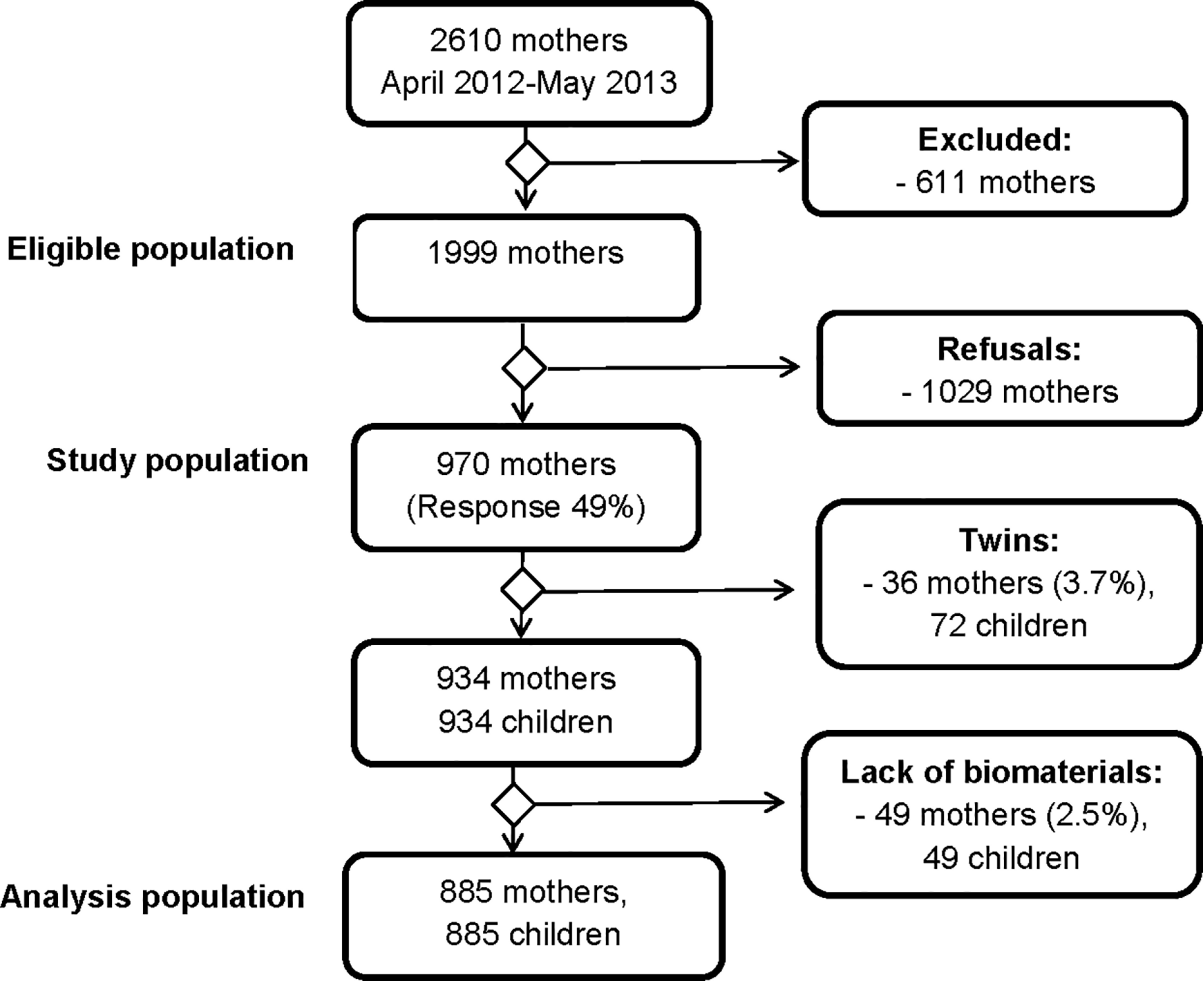
Flow chart of study population and final analysis population.

In the Ulm SPATZ Health Study, newborns and their mothers were recruited from the general population during their hospital stay after delivery in the University Medical Center Ulm, Southern Germany, between 04/2012 and 05/2013. Exclusion criteria were outpatient delivery, maternal age <18 years, insufficient knowledge of the German language, stillbirth, and transfer of the newborn to intensive care immediately after delivery (details in Fig 1). The overall response was 49%. The analysis population included 885 newborns of 885 mothers. Further details can be found elsewhere [13]. Written informed consent was available for all participants. The study was approved by the ethics board of Ulm University (No. 311/11).

### Data collection

Demographic data was collected using a self-administered maternal questionnaire during the hospital stay following delivery. This included detailed questions about family demographics, socioeconomic status, housing and living conditions, as well as medical history, health status, smoking and maternal alcohol consumption (each during pregnancy). Clinical data related to the child’s delivery were obtained from electronic hospital records. Further clinical data related to the mother’s pregnancy were additionally obtained from the routine paper records or prenatal and natal care, which obstetricians in Germany are required to issue to their patients when pregnancy is clinically established and generally updated at each clinical visit during pregnancy.

Maternal body mass index (BMI) at detection of pregnancy was calculated based on weight measured during the mother’s first appointment at which pregnancy was clinically established. BMI was calculated as body weight (kg) divided by body height^2^ (m) and classified into underweight, normal weight, overweight, or obese according to the WHO classification (< 18.5, ≥ 18.5 to < 25, ≥ 25 to < 30, ≥ 30 kg/m^2^). Information on maternal hypertension and diabetes was derived from the maternal questionnaire, electronic medical charts, or using information of the mother’s routine paper records of prenatal and natal care (“Mutterpass”).

### Laboratory analysis

#### Post-delivery maternal serum and cord blood parameters

Maternal blood was collected in a S-Monovette 7.5 ml serum-gel tubes (Sarstedt, Nümbrecht, Germany) during a routine blood withdrawal the day after delivery, centrifuged, and stored long-term at -80°C until further analysis. Cord blood was also collected in a S-Monovette 7.5 ml serum-gel tubes (Sarstedt, Nümbrecht, Germany) shortly after delivery, centrifuged, and stored at -80°C.

Serum uric acid values were measured photometrically with a Cobas 6000 from Roche Diagnostics (Mannheim, Germany, with reagent uricase, enzymatic test, coefficient of variation (CV) 1.3%). This method is standardized against ID/MS. Cystatin C was determined by immunonephelometry on a Behring Nephelometer (BN) II (Siemens, Erlangen, Germany) (CV low concentrations 3.3, high conc. 2.0%). Adiponectin and leptin concentrations in maternal and cord blood serum were determined by a commercially available ELISA (R&D Systems, Wiesbaden, Germany), high sensitivity CRP (hs-CRP, Immunodiagnostik AG, Bensheim, Germany, CV low conc. 5.4%, high conc. 4.0%) and retinol-binding protein 4 (RBP4), measured by immunonephelometry on a BNII (Siemens, Erlangen, Germany, CV 4.3%). HbA_1_c, HDL, triglyceride and insulin were measured according to routine methods in the university laboratory.

#### Hair-cortisol measurement

Cortisol concentrations were determined in the scalp-near 3 cm maternal hair segment, assuming to reflect cumulative cortisol secretion over the period of the preceding three months, i.e., mainly the last trimester of pregnancy. The analytical process for determination of hair cortisol was described in details elsewhere [14]. Briefly, mothers with HCC < 0.09 and > 90.0 pg/mg were not considered for analysis due to uncertainties in the analytical procedure [15]. All laboratory measures were done in blinded fashion.

### Renal function and outcome definition

Glomerular filtration rate was estimated using the cystatin C based CKD-Epi equation [16] [17] because creatinine-based measurements are suboptimal during pregnancy as they underestimate GFR [18]. Birth weight and length were recorded immediately after birth and taken also from the routine paper records of prenatal and natal care. Small for gestational age was defined as the length-related birth weight < 10th percentile. Large for gestational age was defined as the length-related birth weight > 90th percentile based on a German reference population [19].

### Definition of covariates

Variables considered as potential confounders were maternal age at delivery (in years), maternal nationality (German, other), maternal duration of school education (>11 years, ≤11 years), maternal BMI (kg/m^2^), smoking during pregnancy (yes, no), mode of delivery (vaginal, Cesarean), first parity (yes, no), newborn's gender (female, male), maternal alcohol consumption during pregnancy (no, yes), maternal diabetes (yes, no), hypertension before pregnancy (yes, no).

### Statistical analyses

The main characteristics of the study population were described. In order to assess whether the study population was representative for the full study cohort, 95% confidence intervals were calculated for proportions of categorical variables and means of continuous variables for all demographic characteristics of the full cohort. Demographic proportions and means of the corresponding variables within the study population were then compared to the confidence limits among the full study cohort. Then the association of various sociodemographic and medical characteristics with the distribution of SUA and cystatin C were presented and quantified by the non-parametric Kruskal-Wallis chi-square test. The association of maternal SUA and cystatin C with different maternal as well as neonatal biomarkers were analyzed by means of a Spearman`s partial correlation coefficient after adjustment for maternal age.

The association between SUA and cystatin C (both in quartiles as well as continuous measure after log-transformation) with risk for SGA as well as with LGA was quantified by means of multivariable logistic regression after adjustment for maternal age at delivery, and also additionally after adjustment for maternal nationality, education, maternal BMI, diabetes, hypertension before pregnancy, smoking during pregnancy, alcohol consumption during pregnancy, mode of delivery, first parity, maternal HbA1c shortly after delivery, and newborn's gender. In addition association between continuous cystatin C and odds ratio for SGA, respectively LGA, was modelled using cubic restricted splines with knots at the 10th, 50th, and 90th percentile after adjustment for the full set of covariates. All statistical analyses were performed with SAS® 9.4 (The SAS Institute, Cary, NC, USA).

## Results

Table 1 shows the main characteristics of the full study cohort (n = 934) and the study population with measurements of SUA and cystatin C (n = 885) which were included in the final analysis dataset. Most of the mothers were of German nationality (84.9%) and were between 26 and 35 years of age at delivery. Maternal BMI at detection of pregnancy (median 8^th^ week, interquartile range 7 to 10) was between 18.5 and less than 25.0 kg/m^2^ for 59.8% of mothers in the study population, 22.3% were overweight and 12.8% were obese. There were only marginal differences between the total and the study population. SUA serum levels showed a mean of 293.0 μmol/L (interquartile range (IQR) 255.0–340.0) and cystatin C showed a mean of 0.87 mg/L (IQR 0.77–1.00).

**Table 1:** Characteristics of the study population.

	Total Population (n = 934)			Study population (n = 885)	
Variable	N	% or mean	(95% CI)	n	% or mean
**Maternal nationality**					
Germany	788	84.4	(82.0; 86.7)	751	84.9
Other	136	14.6	(12.3; 16.8)	126	14.2
Missing	10	1.1	(0.4; 1.7)	8	0.9
**Maternal education (years)**					
<9 years education	90	9.6	(7.7; 11.5)	82	9.3
10 to 11 years education	280	30.0	(27.0; 32.9)	267	30.2
> 11 years education	545	58.4	(55.2; 61.5)	518	58.5
Missing	19	2.0	(1.1; 2.9)	18	2.0
**Maternal age at delivery (years)**					
< = 25	60	6.4	(4.9; 8.0)	58	6.6
26–35	643	68.8	(65.9; 71.8)	609	68.8
> = 36	231	24.7	(22.0; 27.5)	218	24.6
**Maternal BMI at gynecological detection of pregnancy**					
Underweight (BMI < 18.5)	22	2.4	(1.4; 3.3)	20	2.3
Normal (18.5 < = BMI < 25.0)	552	59.1	(55.9; 62.3)	529	59.8
Overweight (25.0 < = BMI < 30.0)	209	22.4	(19.7; 25.0)	197	22.3
Obese (BMI > 30.0)	120	12.8	(10.7; 15.0)	113	12.8
Missing	31	3.3	(2.2; 4.5)	26	2.9
**Smoking during pregnancy**					
No	844	90.4	(88.5; 92.3)	801	90.5
Yes	70	7.5	(5.8; 9.2)	66	7.5
Missing	20	2.1	(1.2; 3.1)	18	2.0
**Prior parity**					
0 births	497	53.2	(50.0; 56.4)	474	53.6
> = 1 birth	436	46.7	(43.5; 49.9)	410	46.3
Missing	1	0.1	(0.0; 0.3)	1	0.1
**Mode of delivery of current birth**					
Vaginal (spontaneous or assisted)	697	74.6	(71.8; 77.4)	661	74.7
Cesarean (elective or emergency)	236	25.3	(22.5; 28.1)	223	25.2
**Newborn's gender of current birth**					
Male	494	52.9	(49.7; 56.1)	470	53.1
Female	440	47.1	(43.9; 50.3)	415	46.9
**Maternal alcohol consumption during pregnancy**					
No alcohol consumed	281	30.1	(27.1; 33.0)	260	29.4
Alcohol consumption reported	642	68.7	(65.8; 71.7)	615	69.5
Missing	11	1.2	(0.5; 1.9)	10	1.1
**Maternal diabetes ever diagnosed or medication used during pregnancy**					
No	826	88.4	(86.4; 90.5)	781	88.2
Yes	108	11.6	(9.5; 13.6)	104	11.8
**Hypertension before pregnancy**					
No	895	95.8	(94.5; 97.1)	848	95.8
Yes	27	2.9	(1.8; 4.0)	26	2.9
Missing	12	1.3	(0.6; 2.0)	11	1.2
**Cystatin C-based eGFR** (ml/min/1.73 m^2^) median (IQR)	887	97.4	(80.9–110.7)	884	97.5
**CKD stage by eGFRcysC** (ml/min/1.73 m^2^)					
none	192	24.2	(21.2; 27.2)	192	24.3
1 (90+)	267	33.7	(30.4; 37.0)	267	33.8
2 (60–89)	298	37.6	(34.2; 40.9)	295	37.3
3 (30–59)	36	4.5	(3.1; 6.0)	36	4.6
4 & 5 (<39)	0	0		0	0
**Uric acid** (μmol/L), median (IQR)	885	293.0	(255;340)	885	293.0
**Cystatin C** (mg/L), median (IQR)	887	0.87	(0.77; 1.0)	884	0.87

Table 2 presents the distribution of SUA and cystatin C with various sociodemographic and medical characteristics of the study population. There were higher median SUA values associated with German nationality, younger maternal age, higher BMI, vaginal delivery, nullipara, alcohol consumption, and history of diabetes and hypertension. Median cystatin C values were higher in nullipara and marginally higher in male newborns.

**Fig 2:**
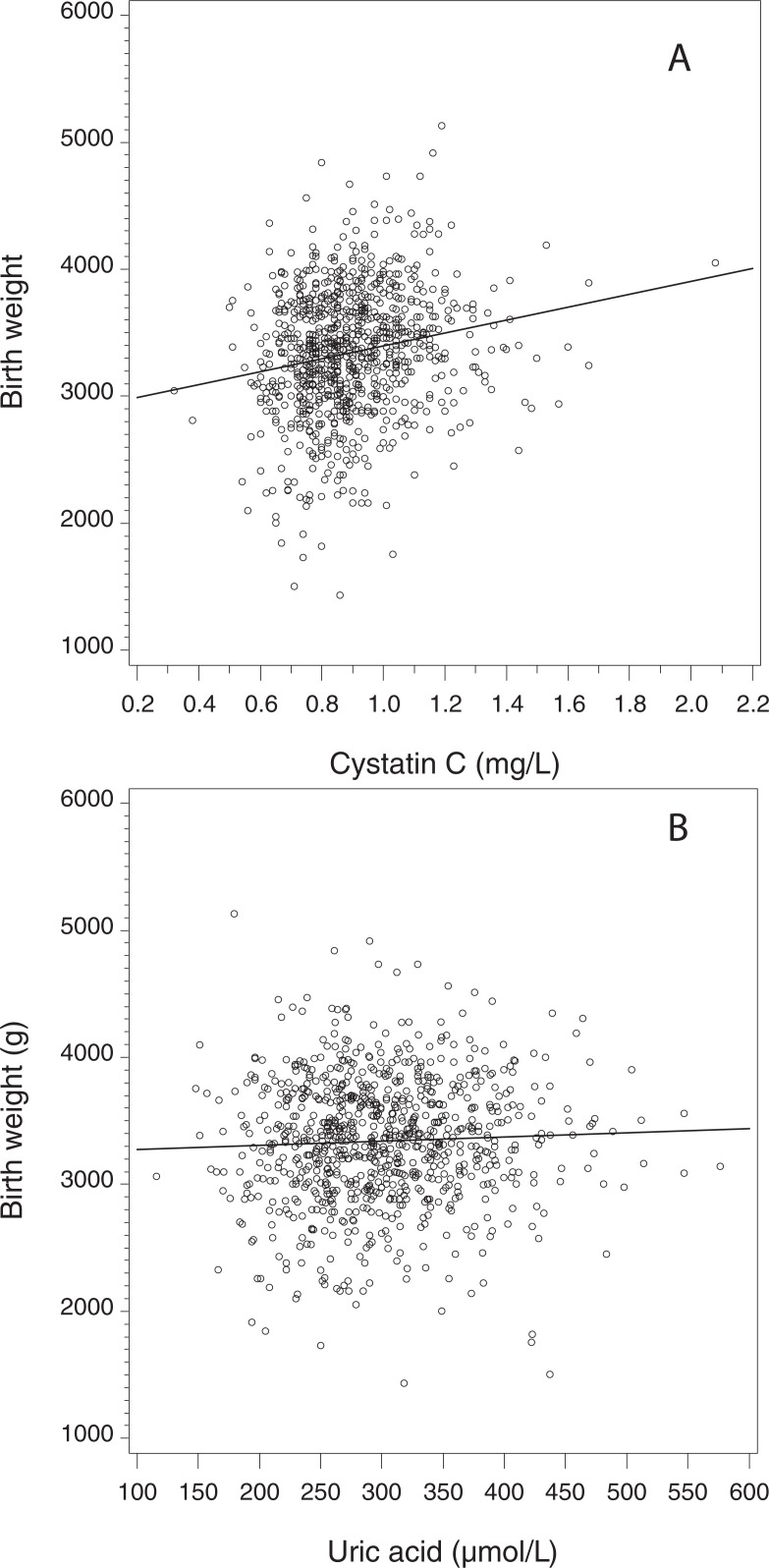
Fig 2 shows the crude relationship between cystatin C and birth weight (panel A; correlation coefficient r = 0.189, p< 0.001) and between SUA and birthweight (panel B; r = 0.056, p = 0.10).

**Table 2:** Distribution of uric acid and cystatin C with various sociodemographic and medical characteristics of the study population.

	n	Uric acid (μmol/L) Median (IQR)	p-value	Cystatin C (mg/L) Median (IQR)	p-value
**Maternal nationality**			**0.038**		0.16
Germany	751	294 (255, 345)		0.87 (0.77, 1.00)	
Other	126	289 (249, 330)		0.86 (0.73, 0.98)	
**Maternal education (years)**			**0.072**		0.94
<9 years education	82	289 (255, 335)		0.86 (0.78, 1.00)	
10 to 11 years education	267	303 (262, 347)		0.87 (0.77, 1.00)	
> 11 years education	518	291 (251, 340)		0.87 (0.77, 1.00)	
**Maternal age at delivery (years)**			**0.021**		0.82
< = 25	58	322 (261, 364)		0.89 (0.79, 0.97)	
26–35	609	294 (254, 343)		0.86 (0.76, 1.00)	
> = 36	218	290 (254, 325)		0.87 (0.78, 0.99)	
**Maternal BMI at gynecological detection of pregnancy**			**<0.0001**		0.65
Underweight (BMI < 18.5)	20	259 (238, 326)		0.87 (0.73, 1.02)	
Normal (18.5 < = BMI < 25.0)	529	287 (248, 327)		0.86 (0.76, 1.00)	
Overweight (25.0 < = BMI < 30.0)	197	304 (265, 350)		0.88 (0.79, 0.98)	
Obese (BMI > 30.00)	113	325 (273, 377)		0.87 (0.78, 1.00)	
**Smoking during pregnancy**			0.54		0.65
No	801	293 (255, 341)		0.87 (0.77, 1.00)	
Yes	66	305 (259, 337)		0.88 (0.78, 1.03)	
**Mode of delivery of current birth**			**0.004**		0.14
Vaginal (spontaneous or assisted)	661	297 (256, 343)		0.87 (0.77, 1.00)	
Cesarean (elective or emergency)	223	281 (250, 332)		0.85 (0.76, 0.99)	
**Prior parity**			**<0.0001**		**0.0011**
0 births	474	305 (262, 355)		0.88 (0.78, 1.02)	
> = 1 birth	410	279 (247, 325)		0.85 (0.76, 0.97)	
**Newborn's gender of current birth**			0.61		**0.054**
Male	470	293 (255, 341)		0.88 (0.77, 1.02)	
Female	415	293 (254, 340)		0.85 (0.77, 0.97)	
**Maternal alcohol consumption during pregnancy**			**0.020**		**0**.08
No alcohol consumed	260	290 (243, 340)		0.86 (0.76, 0.97)	
Alcohol consumption reported	615	296 (257, 343)		0.88 (0.77, 1.01)	
**Maternal diabetes ever diagnosed or medication**			**0.025**		0.38
No	781	291 (254, 340)		0.87 (0.77, 1.00)	
Yes	104	313 (266, 352)		0.89 (0.78, 1.02)	
**Hypertension before pregnancy**			**0.025**		0.60
No	848	292 (255, 340)		0.87 (0.77, 1.00)	
Yes	26	327 (286, 388)		0.84 (0.76, 0.99)	

As displayed in Fig **2** the correlation between cystatin C and birthweight was r = 0.189 (p<0.001) (A) and between uric acid and birth weight r = 0.056 (p = 0.10) (B).

The associations of maternal SUA and cystatin C with different maternal biomarkers after adjustment for maternal age are shown in Table 3. Except for adiponectin we found a clear relationship of increased SUA with almost all maternal cardiometabolic biomarkers in an unfavorable direction. Except for adiponectin, insulin, and leptin, this was also seen in a similar fashion for cystatin C.

**Table 3:** Association of maternal uric acid and cystatin C serum values with different maternal biomarkers after adjustment for age of mother (Spearman`s partial correlation coefficient (r, p-value)).

				Tri-	Adipo-				Hair	Cystatin	Uric
	Insulin	HBA1C	HDL	glyceride	nectin	Leptin	Hs-CRP	RBP4	cortisol	C	acid
**Maternal**	0.089	0.132	-0.080	0.142	-0.017	0.107	0.093	0.071	0.088	0.403	1.00
**uric acid**	0.010	<0.0001	0.020	< .0001	0.63	0.002	0.007	0.04	0.010	< .0001	
**Maternal**	-0.038	0.199	-0.072	0.0869	0.051	0.037	0.081	0.073	0.072	1.00	0.403
**cystatin C**	0.27	< .0001	0.036	0.012	0.137	0.28	0.018	0.034	0.038		< .0001

Table 4 then shows the associations of uric acid and cystatin C with different biomarkers from cord blood (i.e., from newborns circulation) after adjustment for age of mother. Strong associations of both maternal SUA and cystatin C are seen with newborns uric acid, creatinine (which is crossing the placenta), hs-CRP and leptin cord values.

**Table 4:** Association of maternal uric acid and cystatin C serum values with different biomarkers from cord blood after adjustment for age of mother (Spearman`s partial correlation coefficient (r, p-value)).

	Uric acid	Creatinine	Adiponectin	hsCRP	Insulin	Leptin
	cord blood	cord blood	cord blood	cord blood	cord blood	cord blood
**Maternal**	**0.865**	**0.434**	**-0.042**	**0.172**	**-0.049**	**0.142**
**uric acid**	< .0001	< .0001	0.25	< .0001	0.18	< .0001
**Maternal**	0.351	0.446	0.004	0.098	-0.002	0.107
**cystatin C**	< .0001	< .0001	0.92	0.007	0.95	0.003

Associations of SUA and cystatin C concentrations (in quartiles) with risk for SGA and LGA in crude and adjusted analysis are shown in Table 5. For both serum markers an inverse relationship of maternal serum concentrations with decreased risk of SGA is visible in age adjusted analysis, which however, only persisted after multivariable adjustment in models for association of cystatin C (OR in top quartile versus bottom 0.35 (95% CI 0.16–0.77), p for trend within categories 0.04,Panel A). Although the point estimates for SUA-categories with LGA were increased when compared to the bottom quartile, the 95% CI were broad and included the null-values (Panel B). In contrast there was a clear increased risk for LGA with increasing quartiles for cystatin C and the OR in top quartile versus bottom was 5.92 ((95% CI 2.27–15.44), p for trend within categories <0.0001).

**Table 5:** Association of uric acid and cystatin C concentrations (categories, quartiles) with risk for small for gestational age (Panel A) and for large for gestational age (Panel B) by means of logistic regression.

	**Results of multivariable analysis**	
Panel A	**Small for gestational age (SGA)**	
	(length-related birth weight < 10^th^ percentile)	
	OR (95% CI) adjusted for maternal age	OR (95% CI) fully adjusted*
**Uric acid**		
Bottom quartile	1 ^referent^	1 ^referent^
Second quartile	0.65 (0.35–1.20)	0.74 (0.38;1.42)
Third quartile	0.65 (0.35–1.19)	0.73 (0.37–1.42)
Top quartile	0.40 (0.20–0.80)	0.51 (0.24;1.08)
P for trend	0.01	0.09
**Cystatin C**		
Bottom quartile	1 ^referent^	1 ^referent^
Second quartile	0.55 (0.30–1.03)	0.60 (0.3;1.18)
Third quartile	0.77 (0.43–1.37)	0.90 (0.48;1.68)
Top quartile	0.29 (0.14–0.62)	0.35 (0.16;0.77)
P for trend	0.005	0.04
Panel B	**Large for gestational age (LGA**)	
	(length-related birth weight > 90^th^ percentile)	
	OR (95% CI) adjusted for maternal age	OR (95% CI) fully adjusted*
**Uric acid**		
Bottom quartile	1 ^referent^	1 ^referent^
Second quartile	1.52 (0.71–3.24)	1.55 (0.69;3.47)
Third quartile	1.72 (0.83–3.6)	1.80 (0.8;4.03)
Top quartile	1.23 (0.55–2.73)	1.14 (0.47;2.77)
P for trend	0.55	0.73
**Cystatin C**		
Bottom quartile	1 ^referent^	1 ^referent^
Second quartile	1.66 (0.64;4.31)	2.44 (0.86;6.91)
Third quartile	2.57 (1.04;6.36)	3.63 (1.34;9.87)
Top quartile	4.29 (1.84;10.03)	5.92 (2.27;15.44)
P for trend	<0.0001	<0.0001

*adjusted for maternal age at delivery, maternal nationality, maternal education, maternal BMI, smoking during pregnancy, mode of delivery, first parity, newborn's gender, maternal alcohol consumption, maternal diabetes, hypertension before pregnancy, maternal HbA1c

**Fig 3:**
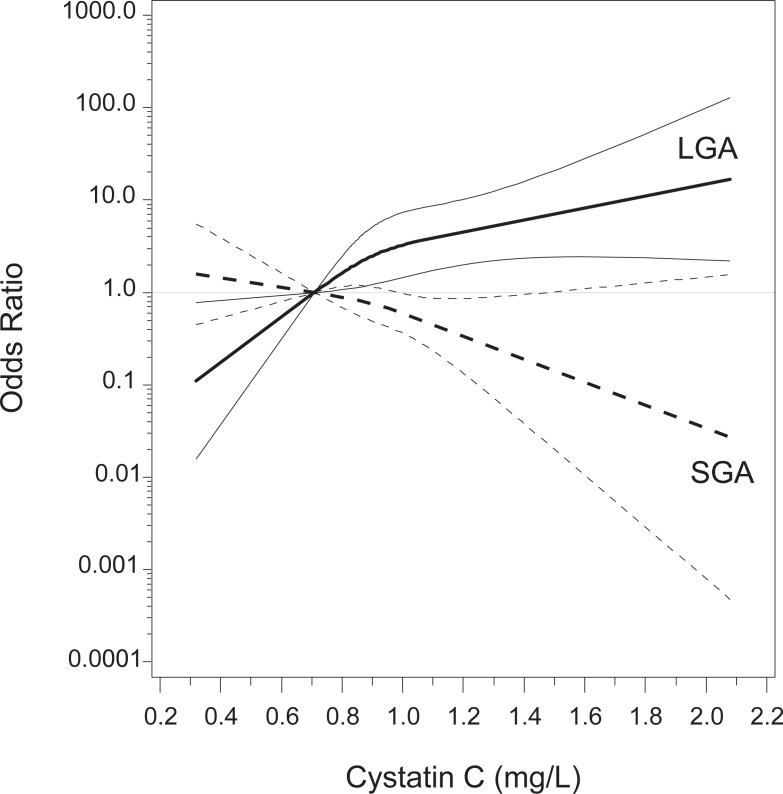
Splines representing OR for C SGA and LGA depending on cystatin C after full adjustment for covariates. The thick lines represent the OR, the thin lines the corresponding 95% confidence bands.

This pattern is also displayed in Fig 3 showing results from the splines representing cystatin C with the OR for SGA and LGA after full adjustment for covariates.

## Discussion

In this group of 885 mother-newborn pairs we found that maternal serum uric acid as well as cystatin C measured shortly after delivery were associated with many maternal as well as with neonatal cardiometabolic risk factors and biomarkers. In addition, we found a positive association of maternal cystatin C serum values with birth weight and a clearly increased risk for LGA with increasing maternal cystatin C values in this population with fairly normal renal function.

So far, there are only limited data from very heterogeneous settings describing the relationships between SUA as well as renal function and birth weight. Laughon et al described an association of SUA with maternal markers of insulin resistance in 263 normotensive women measured around the 20^th^ gestational week, a result in line with our observations [8]. However, contrary to our findings, they reported a positive association with low birth weight. A study from Japan including 40 cases with SGA also described higher SUA and creatinine compared to appropriate-for-gestational age fetuses [9]. In another study from Teheran conducted in 404 normotensive singleton pregnant women, maternal hyperuricemia (defined as a SUA one SD greater than the appropriate value for gestational age) showed an increased risk for preterm birth as well as for SGA (OR 1.28 (95% CI 1.04–2.57) [10]. In contrast, in a study from India including 116 pregnant women, high SUA were associated with low birthweight < 2.5 kg in crude analyses, but no longer after multivariable adjustment. Both markers analyzed in their study (SUA and creatinine), however, were associated with maternal hypertensive disorders in pregnancy [12].

A large study from the Norwegian Mother and Child Cohort including 953 mother and their newborns showed a modest positive association of creatinine–based eGFR estimates (i.e. Cockroft-Gault and MDRD-equation) with infant birth weight (partial correlation coefficient = 0.07), but not when using the recently developed CKD-Epi formula [11]. In addition, a study by Paula and colleagues including 58 hypertensive pregnant women showed an association of a SUA above 357 μmol/L with maternal proteinuria and diastolic blood pressure, but not with fetal outcomes such as SGA [20].

Contrary to the very few existing studies based on creatinine-defined renal function, which suggest an increased risk for SGA, our results clearly indicate an increased risk for LGA with increasing maternal cystatin C value in a population with relatively good renal function. According to a meta-analysis in studies including women with chronic kidney disease (CKD); the risk of adverse outcomes such as intrauterine growth restriction, SGA, or increased mortality was evident [21]. In our study population only few women had signs of CKD (4.6%). Therefore, comparison with CKD-populations is very limited.

As shown by the HAPO study [22] even maternal hyperglycemia less severe than in diabetes might be associated with an increased risk for adverse pregnancy outcomes, including an increased birth weight above the 90^th^ percentile for gestational age. In addition it is known that preeclampsia is associated with glomerular capillary endotheliosis [23], which is then reflected in increased cystatin C serum values. Beside measuring renal function, cystatin C may also be used for the degree of glomerular endotheliosis and reflect a continuum between healthy pregnant women and preeclampsia [24]. Therefore, it may well be, that the association between maternal cystatin C values and LGA in our study may reflect underlying endotheliosis based on an undetected diabetes or hyperglycemia or other causes.

However, correlation of maternal SUA and cystatin C values with maternal as well as cardiometabolic risk factors was relatively clear. Birth weight is an important indicator for a newborn’s health and the intrauterine blood flow supply, as well as, a mirror of the health related conditions of pregnancy. LGA is associated with a higher risk for adiposity, diabetes and cardiovascular diseases in later life [25]. Therefore, the question of whether prenatal exposure to increased maternal SUA and cystatin C within our study population are associated with adverse cardiometabolic phenotypes in upcoming years of childhood warrants further investigation within future follow-up. However, the findings of the current study already highlight the importance to ensure an adequate maternal metabolic profile and good renal function during pregnancy.

Several strengths and limitations should be considered when interpreting the results of our study. Despite several obvious strengths which included a large study population and a prospective design, it is important to consider that renal function during pregnancy is difficult to measure. As we have shown with SUA, selection of biomarkers and time of sampling may be critical to interpretation of results and comparison with other studies. With measurement of cystatin C, we employed a relatively accurate biomarker to assess renal function in comparison to creatinine-based measurements [7]. However, our biomarkers were measured in blood drawn shortly after delivery. Therefore, we have no information about the course during other phases of pregnancy. Beyond HbA_1c_ which covers approximately the last trimester, we do also not have a standardized evaluation of the glycemic, metabolic, and renal situation of the mother during pregnancy and had to rely on documentation of routine examinations. Furthermore, although we could adjust for some of the main risk factors of neonate body composition such as BMI of mother and history of diabetes, we may not have been able to include all relevant factors. And as conditional in observational design we cannot conclude that cystatin C levels are causally related to the outcomes.

Despite these limitations we conclude that maternal increased cystatin C values are associated with an increased risk for LGA within a population with relatively good renal function. SUA was not associated with birth weight, however, both biomarkers were associated with many maternal and neonatal cardiovascular risk markers. These findings highlight the importance to ensure an adequate maternal metabolic profile and good renal function during pregnancy which may result in a better cardiometabolic profile in the offspring and which may subsequently decrease the risk of associated adverse health effects in later life.
